# Wild Halophytic *Phragmites karka* Biomass Saccharification by Bacterial Enzyme Cocktail

**DOI:** 10.3389/fmicb.2021.714940

**Published:** 2021-09-20

**Authors:** Immad Ansari, Uroosa Ejaz, Zainul Abideen, Salman Gulzar, Muhammad Noman Syed, Jing Liu, Wang Li, Pengcheng Fu, Muhammad Sohail

**Affiliations:** ^1^Department of Microbiology, University of Karachi, Karachi, Pakistan; ^2^Department of Biosciences, Shaheed Zulfikar Ali Bhutto Institute of Science and Technology, Karachi, Pakistan; ^3^Dr. Muhammad Ajmal Khan Institute of Sustainable Halophyte Utilization, University of Karachi, Karachi, Pakistan; ^4^Department of Biochemistry, University of Karachi, Karachi, Pakistan; ^5^State Key Laboratory of Marine Resource Utilization in South China Sea, Hainan University, Haikou, China; ^6^Weihai UIC Biotechnology, Inc., Weihai, China

**Keywords:** central composite design, enzyme cocktail, biomass, *Phragmites karka*, saccharification

## Abstract

Biofuel derived from halophytic biomass is getting attention owing to the concerns of energy versus food crisis. The disadvantages associated with edible bioenergy resources necessitate the need to explore new feedstocks for sustainable biofuel production. In this study, biomass from locally available abundant halophytes (*Panicum antidotale*, *Phragmites karka*, *Halopyrum mucronatum*, and *Desmostachya bipinnata*) was screened for saccharification by an enzyme cocktail composed of cellulase, xylanase, and pectinase from *Brevibacillus borstelensis* UE10 and UE27, *Bacillus aestuarii* UE25, *Aneurinibacillus thermoaerophilus* UE1, and *Bacillus vallismortis* MH 1. Two types of pretreatment, i.e., with dilute acid and freeze-thaw, were independently applied to the halophytic biomass. Saccharification of acid-pretreated *P. karka* biomass yielded maximum reducing sugars (9 mg g^–1^) as compared to other plants. Thus, the factors (temperature, pH, substrate concentration, and enzyme units) affecting its saccharification were optimized using central composite design. This statistical model predicted 49.8 mg g^–1^ of reducing sugars that was comparable to the experimental value (40 mg g^–1^). Scanning electron microscopy and Fourier-transform infrared spectroscopy showed significant structural changes after pretreatment and saccharification. Therefore, halophytes growing in saline, arid, and semi-arid regions can be promising alternative sources for bioenergy production.

## Graphical Abstract

Saccharification of phragmites karka.

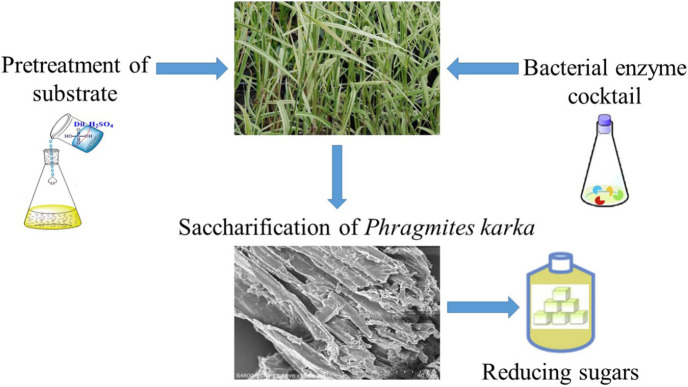

## Highlights

– Halophytic biomass serves as a feedstock energy.
– Dilute acid pretreatment of halophytic plants results in improved saccharification.
– Enzyme cocktail of different bacterial origin is required for maximum saccharification.

## Introduction

About half of the readily available global freshwater is estimated to support the ever-growing world population and associated ecosystems. The population water requirements result in increasing competition for the freshwater, followed by challenges in the context of spread of salinization of agricultural land (Ahmed et al., 2020; Munir et al., 2021). The salinity-resisting potential of halophytes is remarkable as these plant species can survive salinity of up to 5,000 mg l^–1^ (Rozema et al., 2013). These hyper-saline conditions are injurious for the growth of more than 90% of other plant species termed as glycophytes (Rozema et al., 2013). Halophytes do not intrude on prime agricultural lands and are least threatening for the global food supply by protecting good-quality water resources (Ahmed et al., 2020). Moreover, they also reduce the relative cost of crop cultivation and additionally serve as an ideal bio-products manufacturing feedstock (Abideen et al., 2014). Researchers are examining several new emerging halophytes for eco-friendly biofuel feedstocks that can grow in all climate regions. Hereby, halophytes, which produce plenty of biomass in a saline environment, constitute a potential candidate for bioenergy production without compromising human food sources (Smichi et al., 2015). The data about the composition of some halophytes found in the coastline of Sindh and Balochistan, Pakistan, showed ∼25–30% of cellulose and ∼25–30% hemicellulose with less than 10% lignin content (Abideen et al., 2011; Munir et al., 2021), which make them suitable candidates for biorefinery purposes. Among many such plants, *Panicum antidotale*, *Halopyrum mucronatum*, *Desmostachya bipinnata*, and *Phragmites karka* are of particular interest to be used for biomass valorization.

*Panicum antidotale* belongs to the family Poaceae found throughout tropical regions. It can resist salinity and drought conditions, is reported as a useful animal fodder (Khan and Qaiser, 2006), and contains 28% cellulose, 27.97% hemicellulose, 9.1% ash, and 6% lignin (Lima et al., 2019; Munir et al., 2020). A stoloniferous perennial grass, *H. mucronatum*, commonly grows in Asian and African peninsulas on high coastal dunes, has been widely reported for its heavy metal phytoremediation potential in polluted sites (Mujeeb et al., 2020), and contains 38% cellulose, 28.67% hemicellulose, 2% ash, and 5% lignin (Weber et al., 2007; Munir et al., 2020). Another perennial grass, *D. bipinnata*, is composed of 26.67% cellulose, 24.68% hemicellulose, 12.87% ash, and 6.67% lignin (Shah et al., 2017; Munir et al., 2020), is considered as sacred in India, and has been extensively studied for the isolation of medicinally important compounds and for pharmacological properties (Guntur et al., 2018). *P. karka* is reported as the herbaceous woody culms that range from 4 to 10 m in length and 15–25 mm in diameter and is composed of 26% cellulose, 29% hemicellulose, and 10.33% lignin (Munir et al., 2020) and 3.9–8.5% ash (Eller et al., 2020). Moreover, it can tolerate high ranges of salinity and flooding stress with enhanced growth rates (Shoukat et al., 2020). Although Kumari et al. (2017) reported about the utilization of biomass from *H. mucronatum*, *D. bipinnata*, and *P. karka* for the production of fungal enzymes under solid-state fermentation, the studies on the saccharification of these substrates by utilizing bacterial enzyme cocktails do not appear in literature.

Like any other plant material, lignocellulose (LC) biomass from halophytes contains lignin along with fermentable and hydrolyzable components. Thus, to remove lignin and to reduce the crystallinity of cellulose, pretreatment methods are essentially required for effective biomass saccharification with reduced operational costs (Ejaz and Sohail, 2020). The choice of the pretreatment process mainly depends on the type of biomass being valorized and the environmental aspects. Among various chemicals, dilute acid pretreatment has been reported for many glycophytes and halophytic biomass (Smichi et al., 2014), as it offers several advantages, i.e., reduction of thermal degradation of polysaccharides, increased conversion of cellulose to sugars, and low inhibitor concentrations. While the freeze-thaw procedure has been adopted as a milder technique to pretreat halophytic mass (Smichi et al., 2015), it neither releases any toxic chemicals nor requires any special equipment. The pretreated halophytic biomass can be converted into biofuels and other value-added products by biological conversion using enzymatic hydrolysis and microbial fermentation. Use of enzymatic (cocktail) and microbial pretreatment of biomass offers many advantages over chemical approaches, as it is cost-effective and produces few biohazard products (Patel et al., 2019, 2020, 2021). Saccharification of LC through enzymes is an effective green process and relies on various factors, e.g., concentration of the substrate, loading ratio of the enzymes, and optimal enzymatic reaction conditions (Khan et al., 2020). Since a single enzyme with all the desired activities for valorization of LC biomass is not yet available, cocktails composed of various enzymes acting synergistically are employed for this purpose (Song et al., 2016; Tariq et al., 2018). Previously, the enzyme cocktail of xylanase and cellulase was used for the saccharification of corncob, rice, and corn straw (Song et al., 2016). The synergistic action of cellulase and xylanase has largely been explored by utilizing traditional LC substrates, yet the action of enzyme cocktail on halophytic mass has not been reported. In this study, different bacterial species were used to obtain enzyme cocktail. *Aneurinibacillus thermoaerophilus* strain WBS2 was reported as a motile, rod-shaped, aerobic, thermophilic bacterium isolated from the hot spring of India (Acharya and Chaudhary, 2012), whereas *A. thermoaerophilus* UE1 was isolated from soil in Pakistan which was reported to produce thermostable cellulase by fermenting sugarcane bagasse (SB; Ejaz et al., 2020a). Another bacterial species, *Brevibacillus borstelensis*, has been reported for various enzymatic activities including xylanase (Kamsani et al., 2015), amylase (Awasthi et al., 2018), lipase (Tsai et al., 2007), and cellulase by fermenting SB which was isolated from a crocodile pond in Manghopir, Pakistan (Ejaz et al., 2020a), whereas *Bacillus aestuarii* UE25 was reported to produce xylanase (Rashid et al., 2020) and cellulase (Ejaz et al., 2019) by fermenting alkali and ionic liquid-pretreated SB.

This study holds importance as it aimed to screen the abundantly available halophytic plants (*P. antidotale*, *P. karka*, *H. mucronatum*, and *D. bipinnata*) for their potential to be utilized as biomass feedstock. The objective of this study is to saccharify halophytic biomass by using crude enzymatic mixtures obtained from different bacterial species, i.e., a thermophilic bacterium, *B. aestuarii* UE25, *Brevibacillus borstelensis* UE10 and UE27, *A. thermoaerophilus* UE1, and a halotolerant strain, *Bacillus vallismortis* MH 1. Moreover, statistical optimization of enzymatic saccharification was one of the objectives of this work.

## Materials and Methods

### Microbial Strains and Inoculum Preparation

Five bacterial strains (*B. aestuarii* UE25, *Brevibacillus borstelensis* UE10, UE27, *A. thermoaerophilus* UE1, and *B. vallismortis* MH 1) were obtained from the culture collection of the Department of Microbiology, University of Karachi. *B. aestuarii* UE25, *Brevibacillus borstelensis* UE10, UE27, and *A. thermoaerophilus* UE1 produced cellulase, xylanase, pectinase, and amylase, whereas *B. vallismortis* MH 1 produced cellulase and amylase (Table 1). The inoculum was prepared by transferring a single colony in nutrient broth (Oxoid, Thermo Fisher Scientific, Waltham, MA, United States) and incubated for 24 h at 40°C for *B. vallismortis* MH 1 and at 60°C for the other strains. The density of the inoculum was maintained at 0.3 OD_600_.

**TABLE 1:** Endoglucanase (EG), β-glucosidase (BGL), filter paperase (FPase), xylanase, pectinase, and amylase production by bacterial strains.

**Strains**	**Enzyme productivity (IU mL** ^–^ ^1^)^*^					
	**EG**	**BGL**	**FPase**	**Xylanase**	**Pectinase**	**Amylase**
*Aneurinibacillus thermoaerophilus* UE1	9.18	0.45	9.48	10.08	37.5	8.19
*Brevibacillus borstelensis* UE10	15.02	18.91	13.25	21.10	10.71	16.18
*Bacillus aestuarii* UE25	40.92	35.91	10.82	10.27	11.13	16.13
*Brevibacillus borstelensis* UE27	24.92	21.13	10.6	15.29	9.21	18.53
*Bacillus vallismortis* MH 1	9.1	0	0	0	0	7.3

*Insignificant standard deviation.

### Collection of Sugarcane Bagasse and Halophytic Plants

Sugarcane bagasse was collected from a local sugar industry (Pakistan) and ground to the particle size of 300 μ. Halophytic plants, i.e., *P. karka* (PK), *D. bipinnata, H. mucronatum*, and *P. antidotale*, were collected from the saline community of the University of Karachi, Pakistan. The collected plants were air dried in shade and were ground to the size of 100 μ.

### Submerged Fermentation to Obtain Enzyme

For the enzyme production, mineral salt medium (MSM; Mandels and Weber, 1969) with 0.5% (w/v) peptone was used. MSM was either supplemented with 1% (w/v) PK (for *B. vallismortis* MH 1) or with 0.5% (w/v) glucose and 1% (w/v) SB (for the other strains). An inoculum size of 10% (v/v) was added to the MSM. *B. vallismortis* MH 1 was cultivated at 40°C while the other strains were grown at 60°C. After 48 h of incubation, the content was centrifuged for 20 min at 6,000 rpm to get supernatant as a source of crude extracellular enzyme.

### Enzyme Assays

Enzyme assays were performed according to the previously optimized conditions. Enzyme assays for *B. aestuarii* UE25, *Brevibacillus borstelensis* UE10, UE27, and *A. thermoaerophilus* UE1 were performed by using 0.71% of the enzyme substrate (prepared in citrate phosphate buffer, pH 7) at 60°C, while, for *B. vallismortis* MH 1, 0.5% substrate was prepared in sodium citrate buffer (pH 4.8) and assayed at 37°C. The reaction time (15 min) was constant for all the enzymes except for endoglucanase and β-glucosidase 12 min, and for filter paperase 16 min from *B. aestuarii* UE25. Xylanase, pectinase, filter paperase (FPase), β-glucosidase (BGL), endoglucanase (EG), and amylase assay was based on determination of reducing sugars released upon hydrolysis of xylan, pectin, filter paper, salicin, carboxymethyl cellulose, and starch, respectively, by dinitrosalicylic acid method as reported by Miller (1959).

### Pretreatment of Halophytic Plants

Halophytic plants were separately pretreated with dilute acid and by freeze-thaw method (Smichi et al., 2015). For acidic pretreatment, halophytic biomass was added into 1% (v/v) H_2_SO_4_ in 1:20 (w/v) ratio and autoclaved at 121°C. The residues were then washed with water until the pH became neutral and then dried for 48 h at 50°C. For freeze-thaw pretreatment, biomass was frozen for 24 h at −4°C followed by boiling for 15 min at 100°C and then dried for 48 h at 50°C.

### Saccharification of Halophytic Plants

The enzyme preparations from *B. vallismortis* MH 1, *B. aestuarii* UE25, and *Brevibacillus borstelensis* UE27 were standardized as EG, while *Brevibacillus borstelensis* UE10 and *A. thermoaerophilus* UE1 enzymes were standardized as xylanases and pectinases, respectively (Table 1). Saccharification of untreated, freeze-thawed (-FT), and acid-treated (-AT) halophytic plants was carried out by adding 10 U g^–1^ of the enzyme units to the slurry containing 1% (w/v) substrate and 0.2% (w/v) sodium azide in 50 mM sodium citrate buffer (pH 4.8). The temperature employed in this process was 60°C for the enzymes of *A. thermoaerophilus* UE1, *B. aestuarii* UE25, *B. borstelensis* UE10, and *B. borstelensis* UE27, and 40°C for *B. vallismortis* MH 1. Aliquots were drawn at 0 and 24 h, and reducing sugars were estimated by the DNS method (Khan et al., 2020).

### Statistical Optimization of the Saccharification Process

The enzyme cocktail was designed by considering the EG, xylanase, and pectinase activities of the isolates. Based on the initial results, *A. thermoaerophilus* UE1 and *B. borstelensis* UE10 were added as essential components of the enzyme cocktail as these exhibited higher xylanase and pectinase activities while only one among *B. vallismortis* MH 1, *B. aestuarii* UE25, and *B. borstelensis* UE27 was added to the enzyme cocktail. Since the optimal activity of *B. vallismortis* MH 1 differed from the rest of the enzymes, the cocktail containing the *B. vallismortis* MH 1 preparation was therefore investigated at two temperatures, i.e., 40 and 60°C. Finally, the cocktail comprising *A. thermoaerophilus* UE1, *B. borstelensis* UE10, and *B. vallismortis* MH 1 was chosen for the saccharification of AT-PK and hence a central composite design (CCD) was employed to optimize the factors affecting the process. Six factors were investigated including the enzyme units of *A. thermoaerophilus* UE1, *B. borstelensis* UE10, and *B. vallismortis* MH 1 (10, 18, 20, 22, and 30 units), pH (4, 6, 6.5, 7, 9), temperature (30, 38, 40, 42, and 50°C), and substrate concentration (2, 4.4, 5, 5.6, and 8%). The aliquots were collected after 24 h of saccharification for the analysis of reducing sugars.

### Elucidation of Structural Changes in the Substrate

Fourier transform infrared (FTIR) spectroscopy and scanning electron microscopy (SEM) of native PK, acid-pretreated PK, and saccharified PK were carried out by using scanning electron microscopes JSM-6380 A (JEOL, Peabody, MA, United States) and JASCO FT/IR-4200, respectively.

## Results and Discussion

### Saccharification of Halophytic Biomass Pretreated With Acid and Freeze-Thaw

Halophytic biomasses studied in this research are rich in cellulosic and hemicellulosic content (Abideen et al., 2011, 2012). Enzymatic degradation of pretreated halophytic biomass by enzymatic cocktail of thermostable and halophilic bacterial origin has not been studied yet. Use of bacterial crude enzyme cocktail for saccharification of biomass can result in increased sugar yield and is also a cost-effective process. Kumar et al. (2019) used immobilized enzyme cocktail for the saccharification of rice straw and reported 86% saccharification, which shows the efficacy of enzyme cocktail.

For traditional biomass, it is reported that utilization of native substrate without pretreatment yields only 20% glucose (Zhang and Lynd, 2004). In this context, two mild procedures, i.e., freeze-thaw and the use of dilute acid methods, were applied to treat wild halophytic biomasses. In plants, cell wall systems are considered as the primary site of freezing injuries. At a temperature <0°C, a large amount of intracellular ice is formed which damages and ruptures the plasma membrane of plant cells. Serious damage occurs to frozen cells when they are thawed (Smichi et al., 2015). Therefore, to break down cell walls, the freeze-thawing method could be used which increases the accessibility of polysaccharide components to hydrolases. For acidic pretreatment, dilute or concentrated acids are used. Although concentrated acids offer an advantage of working at low temperature (Anwar et al., 2014), dilute acids are preferred to reduce the cost of acid-resistant utensils. Moreover, phenolic compounds, furfural, aliphatic acids, and 5-hydroxymethyl-2-furfural are formed from the degradation of sugars and lignin during pretreatment with a concentrated acid which can inhibit enzymatic hydrolysis substantially (Smichi et al., 2015). Contrarily, pretreatment with dilute acid is performed at higher temperature which dissolves the firm structure of LC biomass. This removes hemicellulose, thereby promoting an increase in enzymatic hydrolysis of cellulosic content (Badiei et al., 2014). Nevertheless, in this study acidic pretreatment was performed with dilute sulfuric acid at 121°C for 15 min. A more efficient saccharification of acid-pretreated biomass from *D. bipinnata*, *P. antidotale*, and *P. karka* was observed as compared to the freeze-thawed substrate by treating with a single enzyme preparation (Table 2). Acid pretreatment also provides certain advantages as dilute acid pretreatment does not require recycling of acid; hence, it is less time consuming and also much safer to use (Moe et al., 2012). The saccharification of biomass from *H. mucronatum* was not seen to yield considerable reducing sugars; therefore, it was not utilized in the subsequent experiments. The probable reason for this limited activity and saccharification is that it can endure as high as 360 mM NaCl equivalent salinity during the mature vegetative stage, which might be inhibit the enzyme activity (Qadri et al., 2019). Yet, the complex interaction of factors such as type of pretreatment, composition of the enzyme cocktail, and chemical composition of the biomass need to be investigated prior to validate any conclusion.

**TABLE 2:** Saccharification of untreated, acid-treated (-AT), and freeze-thawed (-FT) stem and leaves of *Desmostachya bipinnata* (DB), *Halopyrum mucronatum* (HM), *Phragmites karka* (PK), and *Panicum antidotale* (PA).

**Crude enzyme**	**Net reducing sugars (mg g** ^–^ ^1^ of biomass)^*^											
	**PA**	**PA-AT**	**PA-FT**	**PK**	**PK-AT**	**PK-FT**	**HM**	**HM-AT**	**HM-FT**	**DB**	**DB-AT**	**DB-FT**
*A. thermoaerophilus* UE1	0	3.95	4.3	0	13.95	0	7.6	0.3	0.8	0	10.95	0
*B. borstelensis* UE10	0	10.95	5.8	0	14.15	2.45	6.65	0	0.3	0	16.5	0
*B. aestuarii* UE25	2.8	5.3	4.5	0	11.65	0	0.05	0	0	0	11.45	0
*B. borstelensis* UE27	0	10.5	2.7	0	8.45	1.8	0	0	2.6	0	7.8	2.3
*B. vallismortis* MH 1	13.7	17.3	1.65	6.5	6.5	0	9	11.8	1	0	0	2.15

*Insignificant standard deviation.

### Saccharification of Halophytic Biomass by Enzyme Cocktail

Halophytic biomass, like traditional biomasses, is heterogeneous in terms of its composition, and hence, a mixture of enzymes is required to work synergistically for efficient saccharification (Song et al., 2016). It was perceived that a preparation rich in cellulase, xylanase, and pectinase will be required to hydrolyze halophytic mass effectively. Hence, three enzyme cocktails were prepared by mixing the enzyme preparation in different combinations from bacterial strains, i.e., *A. thermoaerophilus* UE1, *B. borstelensis* UE10, and *B. vallismortis* MH 1; *A. thermoaerophilus* UE1, *B. borstelensis* UE10, and *B. aestuarii* UE25; and *A. thermoaerophilus* UE1, *B. borstelensis* UE10, and *B. borstelensis* UE27. As the source of enzyme also affects the efficiency of hydrolysis, considerations were therefore given to the producing strains. *B. borstelensis* UE10 and *B. borstelensis* UE27 and *A. thermoaerophilus* UE1 were previously reported to produce thermostable cellulase by fermenting an agro-industrial waste product, SB (Ejaz et al., 2020a), whereas *B. aestuarii* UE25 was reported to produce cellulase (Ejaz et al., 2019) and xylanase (Rashid et al., 2020) by fermenting alkali- and ionic liquid-pretreated SB. In the present study, except for *B. vallismortis* MH 1, all the strains produced variable titers of multiple hydrolytic enzymes (Table 1). Considering the titers of the enzyme produced, the enzyme preparations of *B. aestuarii* UE25, *B. borstelensis* UE27, and *B. vallismortis* MH 1 were standardized as EG because these strains produced maximum amount of EG as compared to other enzymes (Table 1). The *A. thermoaerophilus* UE1 enzyme was standardized as pectinase, whereas *B. borstelensis* UE10 was standardized as xylanase because these strains produced maximum titers of pectinase and xylanase, respectively (Table 1). This study also possesses another advantage as crude enzyme preparation was used in it, which undermines the need of purification of enzymes for saccharification. Additionally, crude enzyme preparation from bacterial strains contained cellulase, xylanase, pectinase, and amylase except for *B. vallismortis* MH 1 which produced cellulase and amylase only (Table 1). Multienzyme preparation can result in better saccharification. The enzyme preparation from *B. vallismortis* MH 1 was produced using halophytic biomass as substrate rather than a commercial substrate, thus making the process economical. Since acid-pretreated substrates provided better yield of reducing sugars in the initial experiments, the effects of enzyme cocktails were therefore studied by utilizing acid-pretreated halophytic biomass.

The enzyme cocktail containing the enzyme of *B. vallismortis* MH 1 was investigated at two temperatures, 40 and 60°C, considering the optimal activity of the components of the enzyme cocktails. According to the results, the enzyme cocktail comprised preparations from *B. vallismortis* MH 1, *B. borstelensis* UE10, and *A. thermoaerophilus* UE1 yielding 9, 0.5, and 0.3 mg g^–1^ reducing sugars at 40°C from acid-pretreated *P. karka* (PK-AT), acid-pretreated *P. antidotale* (PA-AT), and *D. bipinnata* (DB-AT), respectively (Table 3). The yield was slightly higher when the saccharification process was carried out at 60°C using the same enzyme cocktail (Table 3).

**TABLE 3:** Saccharification of acid-pretreated *Phragmites karka* (PK-AT), *Panicum antidotale* (PA-AT), and *Desmostachya bipinnata* (DB-AT) by different enzyme cocktails.

**Saccharification temperature (°C)**	**Enzyme cocktail**	**Net reducing sugars (mg g** ^–^ ^1^ of biomass)^*^		
		**PK-AT**	**PA-AT**	**DB-AT**
40	UE1^a^, UE10^b^, and MH 1^c^	9	0.5	0.3
60	UE1^a^, UE10^b^, and MH 1^c^	0.6	1.3	4.1
60	UE1^a^, UE10^b^, and UE25^c^	1.1	3.3	2
60	UE1^a^, UE10^b^, and UE27^c^	2.1	1.6	1

*^a^Crude pectinase.*

*^b^Crude xylanase.*

*^c^Crude endoglucanase.*

*Insignificant standard deviation.

The saccharification of PK-AT, PA-AT, and DB-AT by the enzyme cocktail comprising *A. thermoaerophilus* UE1, *B. borstelensis* UE10, and *B. borstelensis* UE27 produced 2.1, 1.6, and 1 mg g^–1^ reducing sugars, respectively (Table 3), whereas, in the case of the cocktail containing *A. thermoaerophilus* UE1, *B. borstelensis* UE10, and *B. aestuarii* UE25, 1.1, 3.3, and 2 mg g^–1^ reducing sugars were produced upon saccharification of PK-AT, PA-AT, and DB-AT, respectively (Table 3). The data in Table 3 show that the highest yield of reducing sugars was obtained when PK-AT was saccharified at 40°C from the enzyme cocktail containing *A. thermoaerophilus* UE1, *B. borstelensis* UE10, and *B. vallismortis* MH 1. In this enzyme cocktail, *A. thermoaerophilus* UE1, *B. borstelensis* UE10, and *B. vallismortis* MH 1 were standardized as pectinases, xylanases, and EGs, respectively. In the enzymatic hydrolysis process, cellulase acts synergistically with other enzymes in the hydrolysis of cellulose (Borges et al., 2014). Although PK did not contain an appreciable quantity of pectin, pectinolytic preparations were also incorporated in enzyme cocktails containing cellulase, as Thite and Nerurkar (2020) reported about the synergistic action of pectinases, xylanases, and cellulases on SB, which also have very low levels of pectin. Pectinases served as accessory enzymes, and ∼1.6- to ∼1.9-fold increases in agro-waste biomass saccharification were obtained by using enzyme cocktail of cellulases, xylanases, and pectinases (Thite and Nerurkar, 2018). In contrast, Luziatelli et al. (2014) emphasized the use of enzyme cocktail comprising cellulase and xylanase for the saccharification of *Tamarix* biomass which resulted in 14.5% saccharification yield.

Previously, saccharification of *Phragmites australis* has been reported (Cotana et al., 2015); however, no study has evidenced the saccharification of *P. karka*. Moreover, soda pulping (under alkaline conditions) of PK has been described to obtain pulp from this plant (Kumar et al., 2013), but there is no available literature about the acid pretreatment of PK. In this study, initial experiments showed that saccharification of PK-AT resulted in 9 mg g^–1^ reducing sugars (Table 3). Therefore, PK is selected for further optimization processes because further investigation is required on this plant to assess its potential as chemical or energy feedstock.

### Central Composite Design for the Saccharification of Acid-Pretreated *Phragmites karka*

**FIGURE 1:**
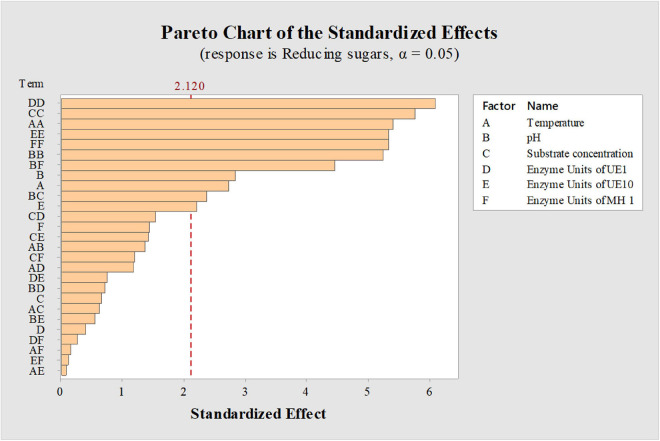
Pareto charts showing the effect of factors on saccharification of acid-pretreated *Phragmites karka.* Dashed line indicates cumulative percentage.

Factors such as substrate concentration, enzyme units, temperature, and pH generally influence the saccharification process (Khan et al., 2020) and regulate the release of reducing sugars. Therefore, optimal conditions need to be determined for higher saccharification yields. However, while working with an enzyme cocktail, it becomes difficult to execute the process under conditions to satisfy each and every component. Indeed, each enzyme in the cocktail is characterized with a different optimal range of working pH and temperature (Kucharska et al., 2019). In the current study, as the components of the enzyme cocktail differed in their optimum temperature for the activity, a balance in operating condition was critically required which was achieved through a statistical design. Parameter optimizations for a process involving multiple components have been proved appropriate by adopting statistical methods (Ejaz et al., 2018; Qadir et al., 2020) as compared to traditional one-factor-at-a-time strategies (Shariq and Sohail, 2018); therefore, a CCD in response surface methodology (RSM) was adopted to optimize the conditions affecting saccharification of acid-pretreated *P. karka* (PK-AT). Six factors (temperature, substrate concentration, pH, and enzyme units of *B. vallismortis* MH 1, *B. borstelensis* UE10, and *A. thermoaerophilus* UE1) were screened by 46 experiments (Table 4). The RSM strategy has also been used previously to optimize the saccharification process of alkali-pretreated *Parthenium* sp. by Pandiyan et al. (2014) and *Eichhornia crassipes* by Das et al. (2015). The factors which were found significant for the saccharification of PK-AT were demonstrated by the Pareto chart (Figure 1). The significance of the design was demonstrated by the *R*^2^ value, i.e., 90.17%.

**TABLE 4:** Central composite design for the saccharification of acid-pretreated *Phragmites karka* by enzyme cocktail.

**Run order**	**Temperature (°C)**	**pH**	**Substrate (%)**	**Enzyme units of**	**Enzyme units of**	**Enzyme units of**	**Net reducing sugars (mg g** ^–^ **^1^**
				**UE1 (IU mL** ^–^ **^1^)**	**UE10 (IU mL** ^–^ **^1^)**	**MH 1 (IU mL** ^–^ **^1^)**	of biomass)^*^
1	38	6	4.4	18	18	18	17.8
2	42	6	4.4	18	18	22	10.3
3	38	7	4.4	18	18	22	16.6
4	42	7	4.4	18	18	18	0
5	38	6	5.6	22	18	18	6.4
6	42	6	5.6	22	18	22	0
7	38	7	5.6	22	18	22	22.7
8	42	7	5.6	22	18	18	3
9	38	6	5.6	18	22	18	16
10	42	6	5.6	18	22	22	14.2
11	38	7	5.6	18	22	22	32.8
12	42	7	5.6	18	22	18	11.2
13	38	6	4.4	22	22	18	22.4
14	42	6	4.4	22	22	22	8
15	38	7	4.4	22	22	22	21
16	42	7	4.4	22	22	18	6
17	40	6.5	5	20	20	20	40
18	38	6	5.6	18	18	22	0
19	42	6	5.6	18	18	18	0
20	38	7	5.6	18	18	18	2
21	42	7	5.6	18	18	22	7.3
22	38	6	4.4	22	18	22	3.8
23	42	6	4.4	22	18	18	0
24	38	7	4.4	22	18	18	6.7
25	42	7	4.4	22	18	22	4.4
26	38	6	4.4	18	22	22	2.5
27	42	6	4.4	18	22	18	8.1
28	38	7	4.4	18	22	18	2
29	42	7	4.4	18	22	22	4.5
30	38	6	5.6	22	22	22	2.5
31	42	6	5.6	22	22	18	1
32	38	7	5.6	22	22	18	3
33	42	7	5.6	22	22	22	7
34	40	6.5	5	20	20	20	15.2
35	30	6.5	5	20	20	20	14.8
36	50	6.5	5	20	20	20	8.6
37	40	4	5	20	20	20	6
38	40	9	5	20	20	20	22.4
39	40	6.5	2	20	20	20	8.1
40	40	6.5	8	20	20	20	4.8
41	40	6.5	5	10	20	20	1.1
42	40	6.5	5	30	20	20	1.9
43	40	6.5	5	20	10	20	10
44	40	6.5	5	20	30	20	15.4
45	40	6.5	5	20	20	10	11.4
46	40	6.5	5	20	20	30	14

*Insignificant standard deviation.

According to the findings in this study, it was observed that the enzyme units, pH, and temperature were significant factors that affected the saccharification of PK-AT (Supplementary Table 1 and Figure 1). Previously, improved hydrolysis of the substrate has been reported with high enzyme loading, probably due to increased saccharification rates (Chen et al., 2007). In this study, a significant effect on the amount of reducing sugars has been observed with the enzyme units >20, whereas >6% substrate negatively influenced the amount of reducing sugars. Also, enzymatic activity might have been hindered by improper mixing due to high substrate load and resulted in less saccharification (Chen et al., 2013). Impurities in enzymatic extract can also interfere with the polysaccharide hydrolysis by inhibiting the formation of the enzyme–substrate complex. Smichi et al. (2014) showed that high concentrations of reducing sugars were obtained after the hydrolysis of acid-pretreated *Juncus maritimus* with a low volume of enzymatic preparation. Here, the maximum effect on saccharification was observed due to the change in pH toward neutral. This might be due to enzyme cocktail adaptability for the pH range from 5 to 7. According to observations, maximum saccharification was seen at 40°C, while further temperature elevation resulted in decreased production of reducing sugars. It is postulated that loss of enzyme activity induced by thermal inactivation might be one of the factors leading to the decrease in saccharification efficiency upon temperature elevation (Karki et al., 2011).

**FIGURE 2:**
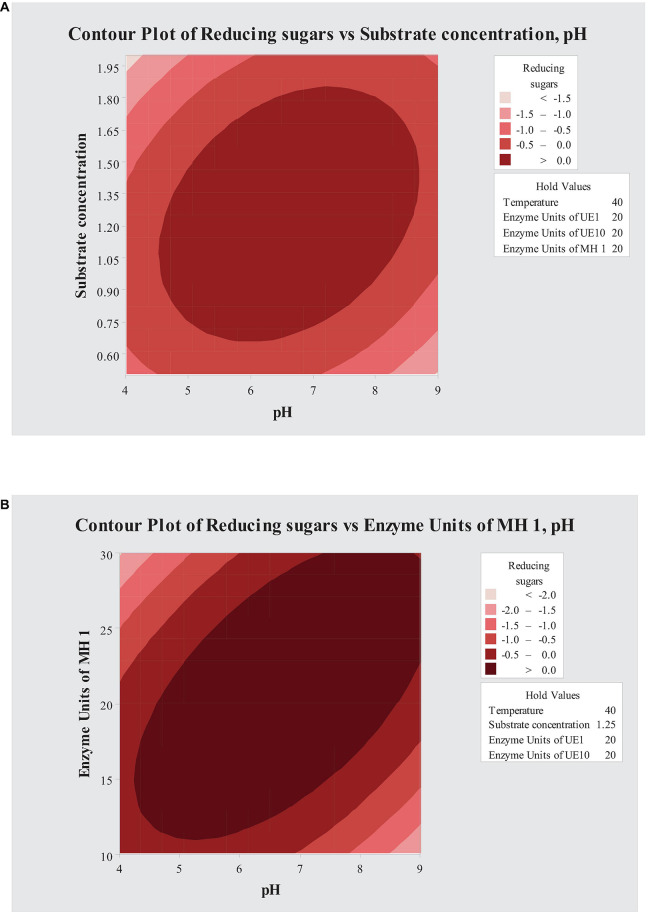
Contour plots showing interaction of **(A)** pH and substrate concentration and **(B)** pH and enzyme units of MH 1.

Contour plots were used to study the interacting effects of variables on saccharification of PK-AT (Figure 2). For the saccharification process, the interaction of pH with *B. vallismortis* MH 1 enzyme units and substrate concentration was found significant (Figure 2).

After the analysis of CCD, software predicted 49.8 mg g^–1^ reducing sugar amount when 5.088% of the substrate was saccharified with 20.30 U of *B. borstelensis* UE10, 20.91 U of *B. vallismortis* MH 1, and 20 enzyme units of *A. thermoaerophilus* UE1, at 40°C and at pH 6.7. According to the experimental results, consistency was observed in the experimental value (40 mg g^–1^) with the predicted value. The positive correlation between experimental and predicted values represented an accurate explanation of optimization of saccharification of PK-AT conditions by RSM. The fact that reducing sugar formation increased by 4.4-fold after optimization as compared to that under unoptimized conditions is also clearly manifested. This yield was much higher than that obtained (5 mg g^–1^) by Khan et al. (2020) where 10 U of crude cellulase preparation was used for the saccharification of SB. Furthermore, crude cellulase preparation by *Bacillus licheniformis* RT-17 resulted in 0.69 and 0.3 mg g^–1^ reducing sugar yield after the saccharification of native SB and alkali-pretreated SB, respectively (Tariq et al., 2018). In another study, enzymatic hydrolysis of the pretreated biomass from a halophyte *Salicornia bigelovii* released high glucose recoveries of up to 90% (Brown et al., 2014). The utmost possible literature survey did not show any report describing the synergistic action of hydrolyzates of *B. vallismortis*, *A. thermoaerophilus*, and *B. borstelensis.* Hydrolysis of biomass into reducing sugars is necessary before other conversion methods into bio-based chemicals and biofuels can take place (Akhlisah et al., 2021). Brust and Cuny (2013) reported a method of conversion of reducing sugars into imidazole heterocycles. Furthermore, the product obtained after the saccharification can also be utilized for ethanol formation (Lu et al., 2012).

### Structural Analysis of Substrate

**FIGURE 3:**
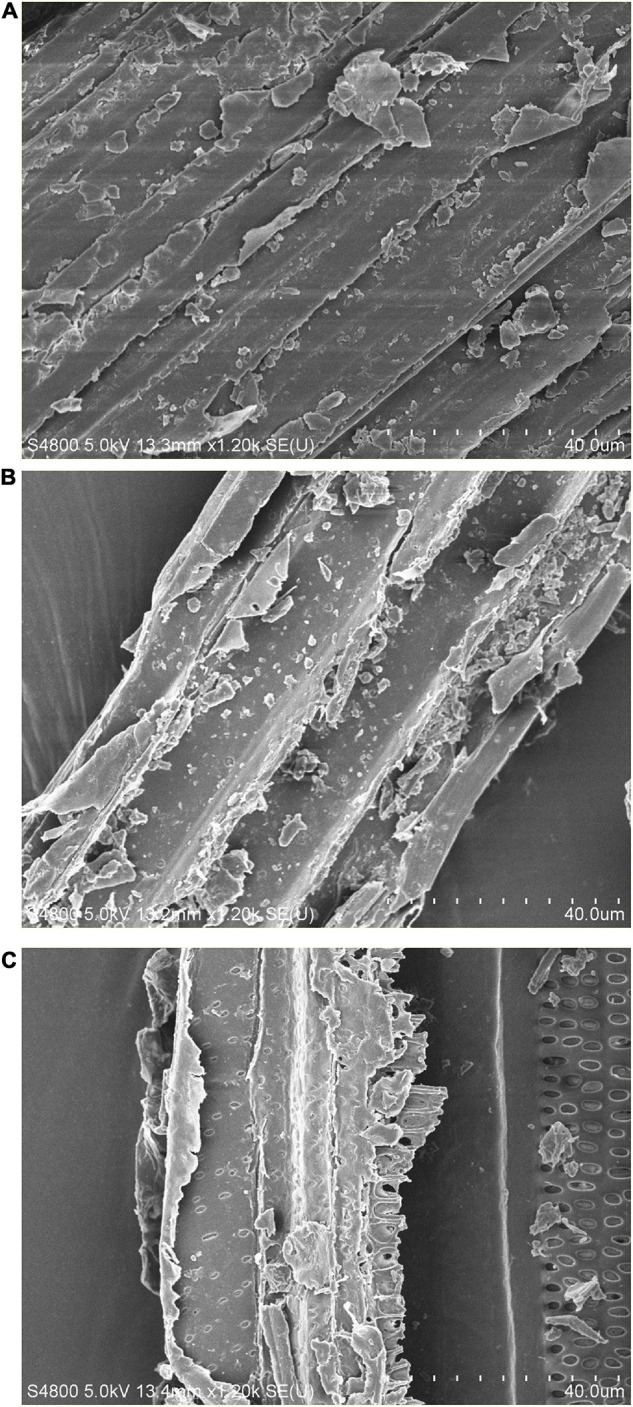
Scanning electron microscopy of **(A)** untreated *Phragmites karka*, **(B)** acid-pretreated *P. karka*, and **(C)** saccharified *Phragmites karka.*

**FIGURE 4:**
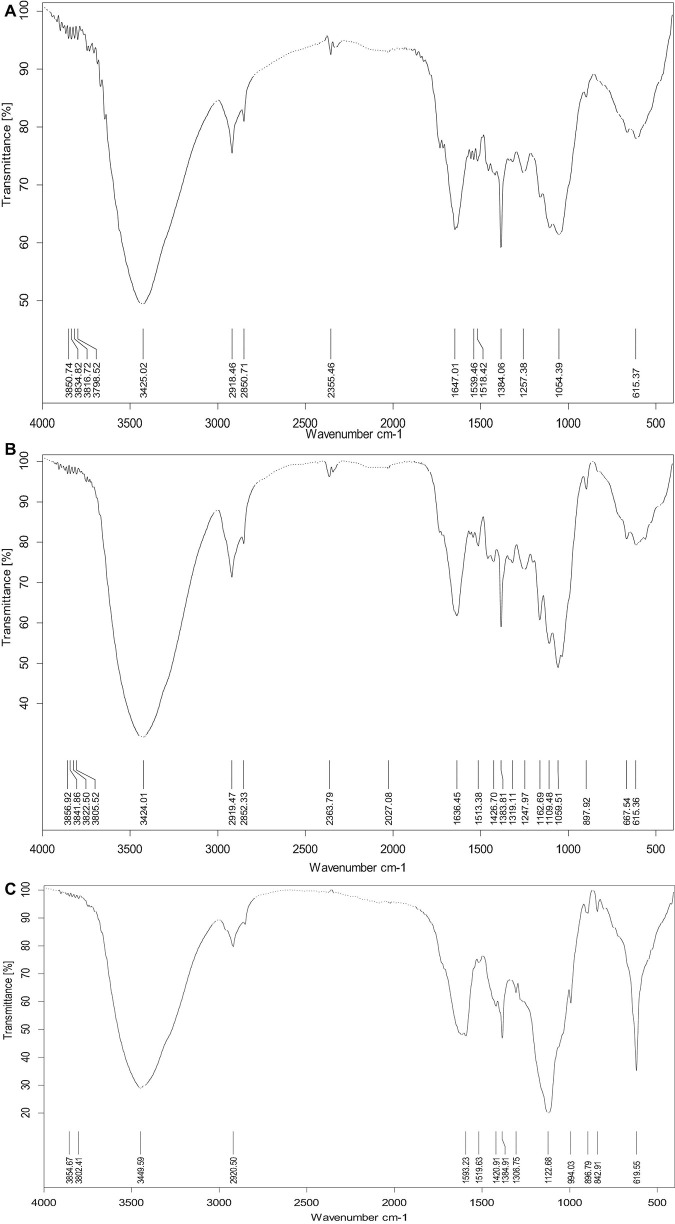
FTIR spectroscopy of **(A)** untreated *Phragmites karka*, **(B)** acid-pretreated *P. karka*, and **(C)** saccharified *Phragmites karka.*

The compact structure of PK was observed in the SEM image (Figure 3A). In general procedures, lignin in LC is removed by chemical pretreatment, which later converts the crystalline cellulose into amorphous type and results in disruption in structure (Ejaz et al., 2020b). In this study, physical disruption has been observed in acid-pretreated PK (PK-AT) through SEM, with damaged surface (Figure 3B). Canilha et al. (2012) reported the separation of pith from fibers caused by pretreatment of LC with dilute H_2_SO_4_. Furthermore, reduction in hemicellulosic and cellulosic content occurred after saccharification, which is evident by complete destruction in structure (Figure 3C). Loosening of the structural matrix and porous structure was observed in saccharified substrate PK-AT (Figure 3C). The results of SEM were further clarified by the images of FTIR (Figure 4).

Fourier transform infrared spectroscopy is generally used as a resourceful technique in order to study the structural changes. In PK-AT, the absence of lignin peaks was observed, particularly at 1,518 and 1,257 cm^–1^, which showed removal of lignin due to dilute acid pretreatment as reported by Singh et al. (2019). The broad-band region at 2,918 cm^–1^ is of C–H stretching vibration which showed a cellulose component (Jayaramudu et al., 2010). The enzymatic digestion of cellulosic content resulted in the peak at 2,918 cm^–1^ for the saccharified substrate. The change in the peak at 1,380 cm^–1^ for the saccharified PK-AT is ascribed to the CH group in a glucose unit, whereas the band at 895 cm^–1^ showed a β-glycosidic linkage (Adel et al., 2011). The saccharification of cellulosic components into reducing sugar and other components was observed as a result of saccharification of PK-AT, contributing change in cellulosic peaks. A similar change was reported by Chandel et al. (2014) in the region between 1,300 and 750 cm^–1^. These observations represent the enzymatic digestion of cellulose.

In addition, we employed principal component analysis (PCA) as another multivariate method to classify FTIR spectra. PCA usually allows the recognition and highlighting characteristics and their correlation to the physicochemical properties of the sample. This method is especially useful in the interpretation of FTIR results, which show band complication and diversity depending on the source of the sample (Szymanska-Chargot and Zdunek, 2013; Matwijczuk et al., 2019). Results showed that native and acid-pretreated substrates differed widely than the saccharified substrate as these were placed on different quadrates, which confirmed that these samples have contrasting properties (Supplementary Figure 1A). Moreover, the peaks representing cellulose and hemicellulose differed significantly that that of lignin, indicating the action of cellulolytic and hemicellulolytic enzymes (Supplementary Figure 1B).

## Conclusion

Halophytic biomass produced under saline environments is a promising feedstock of chemicals and energy. This study recommends that acid-pretreated *Phragmites karka* (PK-AT) can be saccharified by an enzyme cocktail of bacterial origin which consists of 20.30 U of *B. borstelensis* UE10, 20.91 U of *B. vallismortis* MH 1, and 20 enzyme units of *A. thermoaerophilus* UE1, at 40°C and at pH 6.7. The experimental yield of reducing sugars (40 mg g^–1^) was corroborated with the statistical tool, CCD. Structural analysis showed that dilute sulfuric acid pretreatment resulted in significant lignin removal and hydrolysis of cellulosic content of halophytic biomass by the enzyme cocktails. This strategy can be used in future to obtain valuable products from halophytic plants.

## Data Availability Statement

The original contributions presented in the study are included in the article/Supplementary Material, further inquiries can be directed to the corresponding author.

## Author Contributions

IA, UE, and MS: conceptualization. IA, UE, JL, and WL: methodology. IA, UE, ZA, SG, MSy, PF, and MS: investigation, writing, review, and editing. UE: writing of original draft preparation. MS: supervision. ZA, SG, MSy, PF, and MS: resources and funding acquisition. All authors contributed to the article and approved the submitted version.

## Conflict of Interest

PF and MS were employed by Weihai UIC Biotechnology, Inc. The remaining authors declare that the research was conducted in the absence of any commercial or financial relationships that could be construed as a potential conflict of interest.

## Publisher’s Note

All claims expressed in this article are solely those of the authors and do not necessarily represent those of their affiliated organizations, or those of the publisher, the editors and the reviewers. Any product that may be evaluated in this article, or claim that may be made by its manufacturer, is not guaranteed or endorsed by the publisher.
