# Supplementary material for: Wild Halophytic Phragmites karka Biomass Saccharification by Bacterial Enzyme Cocktail
Source: Front Microbiol. 2021 Sep 20;12:714940. doi: 10.3389/fmicb.2021.714940 (PMC8488365; doi:10.3389/fmicb.2021.714940)
Supplement: Supplementary file 1 [file Data_Sheet_1.docx]

**Wild halophytic *Phragmites karka* biomass saccharification by bacterial enzyme cocktail**

**Immad Ansari^s^, Uroosa Ejaz^a,b^, Zainul Abideen^c^, Salman Gulzar^c^, Muhammad Noman Syed^d^, Jing Liu^e^, Wang Li^e^, Pengcheng Fu^e,f^** and **Muhammad Sohail^a,f*^**

**^a^**Department of Microbiology, University of Karachi, Karachi-75270, Pakistan

^b^Department of Biosciences, Shaheed Zulfikar Ali Bhutto Institute of Science and Technology (SZABIST), Karachi-75600, Pakistan

**^c^**Muhammad Ajmal Khan Institute of Sustainable Halophyte Utilization, University of Karachi, Karachi-75270, Pakistan

^d^Department of Biochemistry, University of Karachi, Karachi-75270, Pakistan

^e^State Key Laboratories of Marine Resource Utilization in South China Sea, Hainan University, Haikou 570228, China

^f^Weihai UIC Biotechnology, Inc. 23 Shenzhen Road, Gaocun, Wendeng District, Weihai, Shandong Province, 264408 PR China

*Corresponding author: [msohail@uok.edu.pk](mailto:msohail@uok.edu.pk)

ORCID: 0000-0002-7208-9441

**Running title: Enzyme cocktail for halophytic biomass**

Table S1 P-values and T-values by central composite design.

| **Factor** | **P-value** | **T-value** | **Effect** |
| --- | --- | --- | --- |
| Temperature | 0.015 | -0.014 | Significant |
| pH | 0.012 | 0.014 | Significant |
| Amount of substrate | 0.519 | -0.003 | Non-significant |
| Enzyme Units of UE1 | 0.700 | -0.002 | Non-significant |
| Enzyme Units of UE10 | 0.043 | 0.011 | Significant |
| Enzyme Units of MH 1 | 0.171 | 0.007 | Non-significant |
| Temperature*pH | 0.190 | -1.37 | Non-significant |
| Temperature*Substrate concentration | 0.547 | 0.61 | Non-significant |
| Temperature*Enzyme Units of UE1 | 0.253 | -1.18 | Non-significant |
| Temperature*Enzyme Units of UE10 | 0.936 | 0.08 | Non-significant |
| Temperature*Enzyme Units of MH 1 | 0.877 | 0.16 | Non-significant |
| pH*Substrate concentration | 0.031 | 2.37 | Significant |
| pH*Enzyme Units of UE1 | 0.486 | 0.71 | Non-significant |
| pH*Enzyme Units of UE10 | 0.591 | -0.55 | Non-significant |
| pH*Enzyme Units of MH 1 | 0.000 | 4.45 | Significant |
| Substrate concentration*Enzyme Units of UE1 | 0.144 | -1.54 | Non-significant |
| Substrate concentration*Enzyme Units of UE10 | 0.173 | 1.43 | Non-significant |
| Substrate concentration*Enzyme Units of MH 1 | 0.244 | 1.21 | Non-significant |
| Enzyme Units of UE1*Enzyme Units of UE10 | 0.464 | -0.75 | Non-significant |
| Enzyme Units of UE1*Enzyme Units of MH 1 | 0.794 | -0.27 | Non-significant |
| Enzyme Units of UE10*Enzyme Units of MH 1 | 0.906 | -0.12 | Non-significant |

Figure S1 (a) 2D score plot of principal component analysis. PC1 vs. PC2 (b) PCA loadings according to PCA 1 to represent significant peaks
